# Supplementary material for: Imidazole Processing of Wheat Straw and Eucalyptus Residues—Comparison of Pre-Treatment Conditions and Their Influence on Enzymatic Hydrolysis
Source: Molecules. 2021 Dec 15;26(24):7591. doi: 10.3390/molecules26247591 (PMC8705425; doi:10.3390/molecules26247591)
Supplement: Supplementary file 1 [file molecules-26-07591-s001.zip › molecules-1496594-supplementary.pdf]

Imidazole Processing of Wheat Straw and Eucalyptus Resi-dues—Comparison of Pre-Treatment Conditions and Their In-fluence on Enzymatic Hydrolysis

Pedro M. A. Pereira, Joana R. Bernardo, Luisa Bivar Roseiro, Francisco Gírio, Rafał M. Łukasik*

Laboratório Nacional de Energia e Geologia, I. P., Unidade de Bioenergia e Biorrefinerias, Estrada do Paço do Lumiar 22, 1649-038 Lisbon, Portugal, *e-mail: rafal.lukasik@lneg.pt

Electronic Supplementary Information

Table S1. Composition of solid fractions obtained from wheat straw pre-treated with imidazole.

Conditions		Composition (wt.%)		
T (°C)	t (h)	Cellulose	Hemicellulose	Lignin
130	2	48.2±0.1	25.6±0.5	9.6±0.0
130	4	48.9±1.3	25.4±0.4	9.6±2.4
145	3	50.6±0.7	22.6±0.6	7.9±0.7
160	2	55.1±0.1	24.2±0.7	5.3±0.1
160	4	54.6±0.3	23.8±0.3	4.1±0.0

Table S2. Composition of solid fractions obtained from eucalyptus residues pre-treated with imidazole.

Conditions		Composition (wt.%)		
T (°C)	t (h)	Cellulose	Hemicellulose	Lignin
130	4	50.3±1.2	23.5±0.9	15.9±0.4
145	3	52.7±2.0	26.2±2.0	17.1±0.9
160	4	54.3±0.2	24.4±0.6	16.3±0.3

Table S3. Glucan-to-glucose yield (mol%) of wheat straw pre-treated solids that were produced by imidazole pre-treatment.

Conditions of pre-treatment		Enzymatic hydrolysis time (h)					
T (°C)	t (h)	1	4	6	24	48	72
native		4.3±0.6	11.7±0.1	12.8±0.1	16.1±0.5	18.3±0.5	21.4±0.0
130	2	13.7±0.2	24.7±0.1	31.1±1.0	50.4±1.0	57.8±1.7	62.6±1.5
130	4	15.9±0.2	28.7±0.1	35.1±0.4	57.0±3.8	61.1±3.7	69.8±1.3
145	3	21.8±0.0	39.7±1.4	51.9±1.7	75.4±1.5	83.9±3.3	86.7±1.8
160	2	22.7±1.0	41.2±0.9	52.3±2.4	77.7±0.1	82.4±4.3	84.3±0.8
160	4	32.3±0.3	56.7±0.1	73.6±1.3	87.6±0.8	90.2±1.6	93.3±1.6

Table S4. Glucan to glucose and xylan to xylose saccharification yield (mol%) for 72 h of enzymatic hydrolysis time of wheat straw pre-treated solids as a function of the pre-treatment conditions.

Conditions of pre-treatment		Glucan to glucose	Xylan to xylose
T (°C)	t (h)		
native		21.4±0.5	9.9±0.5
130	2	62.6±1.5	58.6±1.4
130	4	69.8±1.3	60.6±1.9
145	3	86.7±1.8	62.1±1.3
160	2	84.3±0.8	76.2±1.5
160	4	93.3±1.6	77.3±1.0

Table S5. Glucan-to-glucose yield (mol%) of eucalyptus residues pre-treated solids that were produced by imidazole pre-treatment.

Conditions of pre-treatment		Enzymatic hydrolysis time (h)					
T (°C)	t (h)	1	4	6	24	48	72
native		1.3±0.3	2.9±0.5	2.9±0.3	4.1±0.2	5.3±0.4	7.9±1.1
130	4	7.1±0.3	13.1±0.1	15.9±1.0	26.1±0.5	33.9±0.9	40.7±0.9
145	3	7.7±0.0	14.5±1.4	17.5±0.7	29.4±0.9	35.9±1.2	40.6±1.2
160	4	8.3±1.5	17.2±1.2	18.4±1.8	31.0±0.8	36.0±0.6	40.5±0.8

Table S6. Glucan to glucose and xylan to xylose saccharification yield (mol%) for 72 h of enzymatic hydrolysis time of wheat straw pre-treated solids as a function of the pre-treatment conditions.

Conditions of pre-treatment		Glucan to glucose	Xylan to xylose
T (°C)	t (h)		
native		7.9±1.1	4.2±0.2
130	4	40.7±0.9	43.5±1.4
145	3	40.6±1.2	37.9±0.6
160	4	40.5±0.8	41.1±1.9
